# Is There Inflammatory Synergy in Type II Diabetes Mellitus and Alzheimer's Disease?

**DOI:** 10.1155/2012/918680

**Published:** 2012-06-21

**Authors:** Lih-Fen Lue, Cassandra Andrade, Marwan Sabbagh, Douglas Walker

**Affiliations:** ^1^Laboratory of Neuroregeneration, Banner Sun Health Research Institute, 10515 West Santa Fe Drive, Sun City, AZ 85351, USA; ^2^Cleo Roberts Center for Clinical Research, Banner Sun Health Research Institute, 10515 West Santa Fe Drive, Sun City, AZ 85351, USA; ^3^Laboratory of Neuroinflammation, Banner Sun Health Research Institute, 10515 West Santa Fe Drive, Sun City, AZ 85351, USA

## Abstract

Metabolic dysregulation, including abnormal glucose utilization and insulin resistance or deficiency, occurs at an early stage of AD independent of type II diabetes mellitus (T2DM). Thus, AD has been considered as type 3 diabetes. T2DM is a risk factor for AD; the coexistence of these two diseases in a society with an increasing mean age is a significant issue. Recently, research has focused on shared molecular mechanisms in these two diseases with the goal of determining whether treating T2DM can lessen the severity of AD. The progress in this field lends strong support to several mechanisms that could affect these two diseases, including insulin resistance and signaling, vascular injuries, inflammation, and the receptor for advanced glycation endproducts and their ligands. In this paper, we focus on inflammation-based mechanisms in both diseases and discuss potential synergism in these mechanisms when these two diseases coexist in the same patient.

## 1. Introduction

 Alzheimer's disease (AD) and type 2 diabetes mellitus (T2DM) are diseases prevalent in the elderly population. T2DM can increase the risk for developing dementia by 1.5- to 2-fold, and it is considered an important risk factor for AD [1–8]. As the prevalence rate of T2DM is the highest in the age group 65 and older (26.8% in year 2010 according to Center for Disease Controls and Prevention;  http://www.cdc.gov/diabetes/pubs/estimates07.htm), it is a serious concern how T2DM might impact the prevalence rate of AD, and how it might affect the treatment of AD patients. As the mean population age is increasing, both of these two diseases could become much more significant issues. The issue could be further compounded by the epidemic-like phenomenon of obesity that is spreading across all ages [9–11]. At the current annual increase of 0.3–0.6%, there could be 75% of adults that are overweight or obese by 2015 [11]. Obesity is a major risk factor for developing T2DM [2, 12]. Moreover, obesity in middle-age subjects is a negative modifier of T2DM [13]. It has been shown recently that insulin resistance, which is also a risk factor for AD, is associated with lower brain volume and executive function in a large, relatively healthy, middle-aged, community-based cohort [14]. A lack of comprehensive preventive and intervention strategies for these interlinked diseases could lead to a more severe crisis for the healthcare system and the health of the public [15].

 There has been promising progress made in identifying links between T2DM and dementia in the last decade. Special research attention has been directed towards the mechanisms by which T2DM may affect cognitive function and pathogenesis of AD, and towards determining whether treating T2DM might be effective in reducing incidence of AD by modifying AD pathogenesis. The major mechanisms through which T2DM may influence AD include insulin resistance, impaired insulin receptor (IR), and insulin growth factor (IGF) signaling, glucose toxicity, advanced glycation endproducts (AGEs) and the receptor for advanced glycation endproducts (RAGEs), cerebrovascular injury, vascular inflammation, and others [5, 16–20]. There are a number of comprehensive reviews available on insulin resistance and growth factor signaling as molecular mechanisms linking AD and T2DM [8, 16, 17, 21]. Additional discussion focusing on whether there is a causal relationship between AD and T2DM from the studies of epidemiology, clinical trials, and imaging can be found in a review article published in the March issue of Journal of Alzheimer's Disease [18].

The goal of this paper is to focus on a less studied topic: how inflammation-based mechanisms in T2DM might affect AD neuroinflammation and microglial activation. As T2DM and AD both have significant inflammatory components, it is important to assess whether inflammation is synergized when these two diseases coexist. As there has been little research conducted on this aspect, we will review inflammatory mechanisms with respect to each disease and discuss the possibility for these mechanisms to converge.

## 2. Inflammation and Diabetes

 An association of inflammation with T2DM can possibly be demonstrated before clinical diagnosis. This is based on several epidemiological studies that demonstrated greater white blood cell counts or higher levels of inflammatory markers, including C-reactive protein (CRP) and interleukin-6 (IL-6) in healthy middle-aged subjects who later developed T2DM [22–24]. However, not only is chronic inflammation a risk factor for developing T2DM, but it is also an important contributor to the pathogenic mechanisms.

### 2.1. IL-1*β* and Its Receptor

The beta cells from T2DM subjects contain elevated levels of IL-1*β*, a potent pro-inflammatory cytokine, and reduced levels of IL-1 receptor antagonist (IL-1ra) [25]. IL-1ra is a naturally produced molecule that inhibits IL-1*β* activity on its receptor, IL-1 receptor [26]. *In vitro* studies demonstrated that IL-1*β* increased release of insulin by pancreatic islet cells in the presence of high glucose concentration and promoted glucose oxidation [27]. Islet beta cells can be damaged by exposure to IL-1*β*, in a dose- and time-dependent manner [28]. High glucose concentration induced IL-1*β* expression, but reduced expression of IL-1ra, resulting in an imbalance between IL-1*β* and IL-1ra, which impaired insulin secretion and cell proliferation and increased apoptosis [29]. A study in T2DM GK rats has shown that IL-1ra treatment at high dose improved glucose sensitivity, insulin processing, and suppressed inflammation and infiltration of immune cells [30]. The GK rats developed T2DM at a young age and the pancreatic tissues expressed elevated levels of IL-1*β*, and IL-1*β*-driven inflammatory cytokines and chemokines such as tumor necrosis factor-alpha (TNF-*α*), monocyte chemotactic protein-1 (MCP-1), and macrophage inflammatory protein-1alpha (MIP-1*α*), along with abnormal infiltration of macrophages and granulocytes [30]. This study supported that an imbalance between IL-1*β* and IL-1ra leads to pancreatic islet inflammation and release of insulin. Clinical trials using anakinra, a recombinant human IL-1ra, or inhibition of IL-1 receptor signaling has shown effectiveness in correcting beta cells dysfunction and reduced systemic inflammation in T2DM [31, 32]. In fact, IL-1ra is the only anti-inflammatory treatment approved by Food and Drug Administration for T2DM [33].

### 2.2. RAGE and the Ligands

The receptor for advanced glycation endproducts (RAGE), a pattern-recognition receptor, interacts with its ligands resulting in persistent inflammatory responses at sites where the ligands concentrate. These mechanisms have been shown to play a pivotal role in propagation of vascular injuries, a major complication of diabetes [34–37]. The major RAGE ligands in diabetes are advanced glycation endproducts (AGEs), which are derivatives of lipids, proteins, and ribonucleic acids. These are modified by nonenzymatic glycosylation, followed by rearrangement, dehydration, and eventually becoming irreversible cross-linked macromolecules [38, 39]. The amount of these heterogeneous products increases with age, but is further enhanced by diabetes or hyperglycemic conditions [40–42]. Circulating neutrophils can play a role in enhancing the formation of AGE in response to inflammatory activation of the myeloperoxidase system [43]. Diabetes-associated RAGE-AGE interactions induced reactive oxygen species-mediated inflammatory responses in vascular cells (endothelial cells, smooth muscle cells, and pericytes) and mononuclear phagocytes; all of these cells are critically involved in diabetes-associated atherosclerosis, nephropathy, and retinopathy [37, 44–48].

Recent evidence also demonstrated that RAGE is involved in inflammation-based mechanisms of islet cell death. Activation of RAGE by S100B and high mobility group box 1 (HMG1) caused apoptotic death of pancreatic beta cells through an NADPH oxidase-mediated mechanism [49]. The interaction of AGE with RAGE induced apoptosis of islet beta cell and impaired the function of secreting insulin in an *in vitro* study [50]. Inhibition of AGE formation and blockade of RAGE-mediated chronic inflammatory mechanisms are currently considered to be therapeutic strategies for diabetes and diabetes-associated macro- and microvascular complications [51–54].

Human vascular cells express a novel splice variant of the RAGE gene that encodes for a soluble RAGE protein, named endogenous secretory RAGE (esRAGE). The esRAGE protein neutralizes the action of AGE on vascular cells, thus preventing AGE from activating cell-surface (or full-length) RAGE signaling [55]. There is another form of soluble RAGE (sRAGE) that is not generated by alternative splicing; instead, it is a product of catalytic cleavage of membrane bound full-length RAGE by enzymes such as a disintegrin and metalloprotease 10 [56–58]. There was a negative correlation between the expression levels of full-length membrane RAGE and sRAGE expression in monocytes from T2DM [59]. Enhancing sRAGE-associated protective mechanisms are also molecular targets in developing T2DM therapeutics [60].

### 2.3. Other Pattern-Recognition Receptors

Toll-like receptors (TLRs) are pattern-recognition receptors consisting of 12 family members in humans. They are crucial for innate immune functions. Evidence has emerged that some of the TLR members are involved in mediating inflammatory responses in metabolic disorders. TLR2 and TLR4 expressions were elevated in the cell surface of monocytes, derived from patients with metabolic syndrome, and released higher levels of IL-1*β*, IL-6, and Il-8 following lipopolysaccharide stimulation [61, 62]. High glucose increases the expression of TLR2 and TLR4, which can be accentuated by the presence of free fatty acids [63, 64]. These effects were mediated via protein kinase C (PKC)-*α*/PKC-*δ* by stimulation of NADPH oxidase [63]. The inflammatory responses induced by TLR2 and TLR4 are mediated through the activation of NF-*κ*B [65]. TLR4 is upregulated in pancreatic islet cells and a chemokine ligand, interferon-inducible protein (IP)-10 (or CXCL10), was identified to activate this receptor leading to islet cell death [66]. IP-10 can be induced by high glucose through TLR2 and TLR4 [67].

CD36 (oxidized low-density lipoprotein receptor, oxLDL receptor, or scavenger receptor B, MSR-B) is also a pattern recognition receptor which serves as a co-receptor for TLR2 and TLR6 heterodimers, as well as TLR4 and TLR6 heterodimers [68]. High glucose, oxLDL, free fatty acids, and low high density lipoprotein receptors (HDLs) cholesterol concentrations were shown to increase the expression of CD36 in monocytes/macrophages, resulting in vascular oxidative injury, increased leukocyte adhesion, and promoting atherogenesis [69]. Deficiency of CD36 in transgenic mice improves insulin signaling, inflammation, and atherogenesis [70, 71].

## 3. Diabetes and Alzheimer's Disease Pathology

 There have been several studies investigating whether T2DM worsens the hallmark pathology of AD, namely, neuritic plaques and neurofibrillary tangles. In a study involving 143 diabetic and 567 nondiabetic AD patients, no differences were observed between these two groups in A*β* load, neuritic plaque, and neurofibrillary tangle scores [72]. In another study, the presence of diabetes has even been shown to be negatively associated with the abundance of neuritic plaques and neurofibrillary tangles [73]. In line with this finding, Nelson and colleagues observed that although AD patients with diabetes had significantly more infarcts and vascular damage, the plaque scores, as measured by Consortium to Establish a Registry for Alzheimer Disease criteria, were significantly lower [74]. Using biochemical and histological approaches, Sonnen et al. found inconsistent results between biochemical and neuropathological results [75]. Using formic acid to extract detergent-insoluble A*β* from amyloid deposits in superior and medial temporal samples, they found that the concentrations of A*β*42 in formic-acid extract were significantly higher in AD patients without T2DM than in AD patients with T2DM. This was regardless of neuritic plaque scores and neurofibrillary tangle distribution that did not differ between AD cases with and those without T2DM. The same study also investigated whether T2DM leads to more oxidative reactivity and neuroinflammation. The results showed that AD cases without T2DM had significantly higher levels of free-radicals as measured by F_2_-isoprostanes, whereas AD cases with T2DM had significantly greater IL-6 concentrations in cortical tissues than AD without T2DM. It is worth noting that IL-6 is one of three key acute phase proteins shown to be significantly elevated in temporal cortical samples of AD subjects [51]. Neurons in the brain of T2DM patients could be more vulnerable to the toxicity of A*β* due to the defective insulin receptor signaling [76]. Conversely, the defect in insulin receptor signaling could lead to increased production of A*β* and A*β*-induced oxidative damage of the mitochondria [17]. These are among the mechanisms that increase the neuronal degeneration in association with the condition of T2DM.

**Figure 1:**
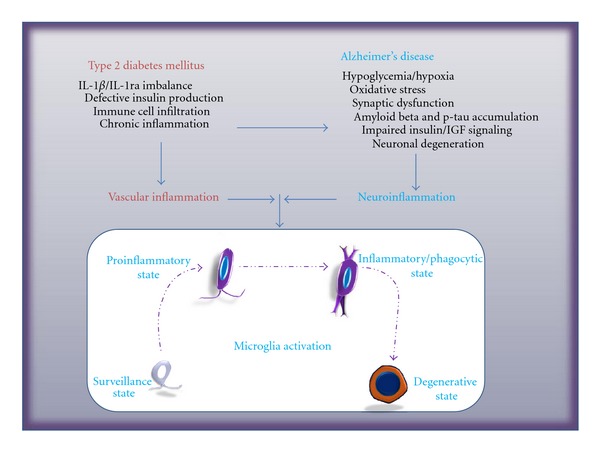
Potential effects of type 2 diabetes mellitus on microglial activation in Alzheimer's disease. Type 2 diabetes mellitus (T2DM) affects the brain with chronic impairment of insulin production and glycaemic control in the periphery. T2DM also causes macro- and microvascular diseases in which inflammation plays a pivotal role. Cerebral microvascular diseases developed from T2DM complications lead to compromised blood-brain barrier function and endothelial cell activation. Microglia can respond to vascular injury and inflammation. Microglial activation is a process of functional and morphological transformation. We propose here that they can be staged as surveillance, proinflammatory, inflammatory, phagocytic, and degenerative states; the transformation depends on the type, distance, potency, and duration of stimulation. We propose that T2DM might promote the activation of microglia through vascular inflammation and the effects on neuronal metabolic dysfunction.

When determining whether T2DM affects the types and development of amyloid plaques, a significant increase in A*β*40-immunoreactive dense plaques, but not in cored plaques, was observed [77]. Dense plaques are considered to be at an earlier stage of maturation, and more toxic than core-only plaques (or burnt-out plaques). Using RAGE immunoreactivity as a marker for oxidatively stressed cells, the authors detected a significant increase in RAGE-immunoreactive cells in the hilus of dentate gyrus in AD cases with T2DM than in AD cases without T2DM [77]. It is intriguing how T2DM might affect the maturation of amyloid plaques. Could this be mediated through its effects on microglial activation? The authors noticed a looser association of activated microglia with dense plaques in AD subjects with T2DM when compared to AD subjects without T2DM. There could be several possible interpretations for this finding. It could suggest that there was an enhanced microglial phagocytic function in AD with T2DM, thus facilitating the removal of amyloid surrounding the amyloid core. This could also be due to the modification of microglia activation state by additional stimuli in AD with T2DM. Previous research has shown the association of primed, enlarged, or phagocytic microglia with amyloid plaques of different maturation stages [78]. When IL-1*α* used as a marker for microglial activation, a greater number of IL-1*α*-immunoreactive microglia were associated with diffuse neuritic amyloid plaques, but they did not associate with nonneuritic dense core plaques [78, 79]. A more detailed analysis is necessary to elucidate whether co-existence of T2DM with AD alters the development of amyloid plaques, as well as the phenotypic and functional characteristics of microglia activation. This would require utilization of various microglial activation markers along with antibodies that can detect A*β*40- or A*β*42-predominant amyloid plaques, and antibodies that can detect neuritic components within the plaques. The potential effects of T2DM on microglia activation during development of AD are proposed in Figure 1.

## 4. RAGE-Mediated Inflammation in AD Brain

 RAGE-mediated mechanisms play crucial roles in the pathogenesis of T2DM and associated vascular complications, but RAGE is also an important cell-signaling receptor involved in various aspects of AD. RAGE is expressed in the brain in neurons, microglia, and astrocytes [80–82]. A*β* is a specific ligand for RAGE, which interacts with the N-terminal domain of RAGE [83]. RAGE expression was elevated in AD pathology-enriched brain regions, including hippocampus and inferior frontal cortex, when compared to cerebellum where AD pathology is limited. RAGE expression was also increased in neurons and microglia in the hippocampus [80, 82]. The interaction of A*β* with neuronal RAGE leads to reactive oxygen species-mediated cellular stress and activation of the transcription faction NF-*κ*B, resulting in increased inflammatory gene and protein expression. For example, elevated secretion of macrophage colony-stimulating factor (M-CSF) and tumor necrosis factor alpha (TNF-*α*) by microglia and BV-2 cells was observed [80, 84]. In experiments using cultures of postmortem human microglia and an *in vitro* A*β* plaque model, A*β*-induced directional migration of microglia was shown to be RAGE-dependent. This was shown by the inhibition of microglial migratory responses to A*β* when RAGE was blocked by anti-RAGE (Fab′)_2_ [80]. The involvement of RAGE-mediated microglial activation in exacerbation of synaptic degeneration, neuroinflammation, and A*β* levels has been illustrated in a study that compared human amyloid precursor protein (APP) single-transgenic mice to double-transgenic mice over expressing the human RAGE gene in microglia along with mutated APP transgene [85, 86]. Enhanced IL-1*β* and TNF-*α* production, increased infiltration of microglia and astrocytes in amyloid plaques, increased levels of A*β*40 and A*β*42, reduced acetylcholine esterase (AChE) activity, and accelerated deterioration of spatial learning/memory were observed in the double-transgenic mice when compared to single transgenic APP or RAGE mice [85]. The involvement of microglial RAGE in driving these consequences was further elucidated in the same study by using signal transduction-defective mutant RAGE [dominant negative (DN)-RAGE] to microglia. The DN-RAGE gene in APP transgenic mice prevented the loss of AChE activity, reduced plaque load, and improved spatial and memory functions [85]. These findings demonstrated that RAGE signaling in microglia played a critical role in promoting inflammatory responses that could lead to increase in A*β* levels and synaptic dysfunction.

 Increased association of AGEs, a RAGE ligand, has been observed in amyloid deposits, and in astrocytes and microglia. This correlated with increased inducible nitric oxide synthase in AD pathology-rich area [87]. The nitric oxide-mediated oxidative mechanisms can mediate the cytotoxicity of AGE [88]. Other RAGE ligands upregulated in AD brains include S100B, S100A9, S100A12, and HMG1 [89, 90]. Although S100B and S100A8 are known as inflammatory cytokines of myeloid phagocytes, their expression by human microglia can be induced by chronic exposure to A*β*1-42 [91].

 Increases in formation of AGE could also result in upregulation of macrophage scavenger receptor CD36. Elevated expression of CD36 correlated with the presence of amyloid deposits, but not the clinical diagnosis of AD. The expression of CD36 by microglia promotes adhesion to fibrillar A*β*, increases oxidative stress and proinflammatory responses, and affects microglial uptake of A*β* [92].

## 5. RAGE, Ligands, and Cytokine Cascade

 One feature that makes RAGE a critical inflammatory receptor is that its expression is increased by its ligands; this creates a positive feedback mechanism that can perpetuate inflammation once it sets off [37, 46, 93, 94]. The amplification of inflammatory consequences can also be further fueled by additional cytokines. For example, in monocytic lineage cells, preexposure to AGE followed by treatment of IL-6 or TNF-*α* can induce release of the RAGE ligands, S100A8 and S100A9 [95]. Preexposure of endothelial cells to AGE has also been shown to increase IL-6, intercellular adhesion molecule-1, vascular adhesion molecule-1, and MCP-1 upon stimulation with S100A8/A9 heterodimers [93]. These findings illustrate how RAGE and its ligands can combine with cytokine-mediated inflammation to exacerbate chronic inflammatory diseases such as AD and T2DM.

As in T2DM, there is a deficiency in the anti-inflammatory function of sRAGE in AD due to a gradual decline in the circulating levels of sRAGE [57, 96, 97]. With this protective function being compromised and with several RAGE ligands elevated, it is possible that the coexistence of AD and T2DM would result in accentuated inflammatory responses, both in the periphery and in the brain. Small molecules that can block RAGE activation or enhance the protective function of sRAGE are a strategy which may be beneficial to both AD and T2DM [98, 99].

## 6. Conclusion

**Figure 2:**
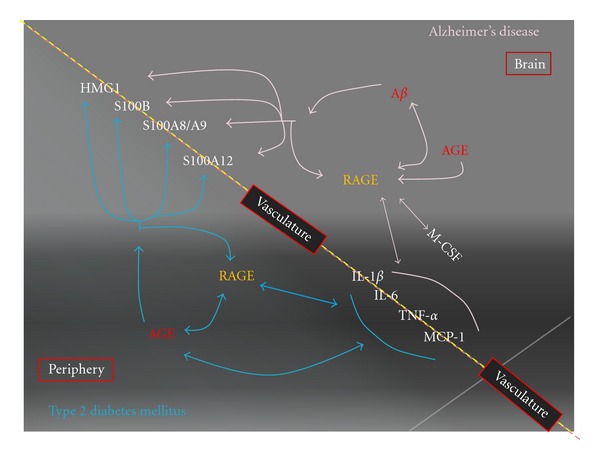
RAGE-driven inflammatory synergy in Alzheimer's disease with type 2 diabetes mellitus. Receptor for advanced glycation endproducts- (RAGEs) mediated inflammatory responses play an important roles in pathogenesis of Alzheimer's disease (AD) and type 2 diabetes mellitus (T2DM). In AD, A*β* is the most prominent ligand that interacts with RAGE leading to inflammatory signaling. The interaction also leads to microglia secretion of M-CSF which can further upregulate the expression of RAGE in microglia. Other inflammatory cytokines and chemokines are also produced upon the activation of RAGE, including IL-1*β*, IL-6, TNF-*α*, and MCP-1. Several of these inflammatory mediators also can modulate the expression of RAGE and its ligands. A number of other ligands are also expressed at elevated levels in the AD brain including AGE, S100A8, S100A9, S100A12, S100B, and HMG1. In T2DM, advanced glycation endproducts (AGEs) are the major ligand. Interaction with RAGE, AGE induces production of other RAGE ligands and inflammatory cytokines and chemokines, which is the major mechanism for propagation of vascular inflammatory injury in T2DM-associated vascular diseases. Thus, RAGE-mediated inflammatory responses might be accentuated when these two diseases coexist in the same patient.

There is strong evidence supporting inflammation as key feature in the brain of AD and in the pancreas of T2DM as summarized in Table 1. A wide range of inflammatory mediators and receptors are involved in these two diseases, although complement activation is a prominent feature in AD, but not in T2DM [100]. The presence of infiltrated lymphocytes is controversial in AD [76, 101]. Therefore, current research findings support the inflammation-based pathogenic mechanisms in both diseases. Although research investigating that T2DM may alter brain inflammation in AD is limited, there is a great possibility that T2DM could accentuate microglial activation, neuroinflammation, and vascular inflammatory/oxidative injury in AD brains through mechanisms mediated by RAGE and other pattern-recognition receptors, and the cascade of cytokine and chemokines.  Figure 2 illustrates the potential of RAGE-centric mechanisms in the central and peripheral systems when both diseases coexist. As microglia play a central role in initiation and propagation of neuroinflammation, and anti-inflammation is one of the preventive and disease modifying strategies for AD, more studies will be needed to characterize the patterns of microglial activation in AD patients with T2DM and AD patients without T2DM.

## Figures and Tables

**Table 1:** Inflammatory responses detected in the brain of Alzheimer's disease and the pancreas of diabetes mellitus.

	Disease-affected brain regions in Alzheimer's disease patients	Pancreas in T2DM patients
Elevated inflammatory markers	Cytokines (e.g., IL-1*α*, IL-1*β*, IL-6, TNF-*α*), chemokines (e.g., IL-8, MCP-1), acute phase proteins (e.g., ACT-1, Serum amyloid P), activated complement proteins (e.g., C3, C5a, C5b-9), and S100B	Cytokines (e.g., IL-1*β*, IL-6, TNF-*α*), chemokines (e.g., IL-8, IP-1, MCP-1, MIP-1*α*), growth factor (G-CSF), S100B, and HMGB1

Immune cell infiltration	Rare presence of lymphocytes or macrophages	Increased macrophages, T-lymphocytes, and granulocytes

Involvement of pattern recognition receptors and major ligands	MSR-A, MSR-B, RAGE, TLR2, TLR4; amyloid beta, AGE	MSR-A, MSR-B, RAGE, TLR2, TLR4: amylin, AGE, IP-10

## Abbreviations

AD: Alzheimer's disease
AChE: Acetylcholine esterase
(ACT-1): Anti-chymotrypsin-1
AGE: Advanced glycation endproducts
APP: Amyloid precursor protein
CRP: C-reactive protein
DN: dominant negative
esRAGE: endogenous secretory receptor for advanced glycation endproducts
G-CSF: Granulocyte-colony stimulating factor
HMG1: High mobility group box
IGF: Insulin growth factor
IR: Insulin receptor
IL: Interleukin
(IL-1ra): Interleukin-1 receptor antagonist
IP-10: Interferon-inducible protein-10
MCP-1: Monocyte chemotactic protein-1
M-CSF: Macrophage colony stimulating factor
(MIP-1): Macrophage inflammatory protein-1
MSR: macrophage scavenger receptor
HDL: High density lipoprotein
(ox-LDL): oxidized low density lipoprotein
PKD: Protein kinase
RAGE: Receptor for advanced glycation endproducts
sRAGE: Soluble RAGE
TLR: Toll-like receptor
TNF-*α*: Tumor necrosis factor-alpha
T2DM: Type 2 diabetes mellitus.
